# The Association Between Alzheimer's Disease-Related Markers and Physical Activity in Cognitively Normal Older Adults

**DOI:** 10.3389/fnagi.2022.771214

**Published:** 2022-03-28

**Authors:** Steve Pedrini, Pratishtha Chatterjee, Akinori Nakamura, Michelle Tegg, Eugene Hone, Stephanie R. Rainey-Smith, Christopher C. Rowe, Vincent Dore, Victor L. Villemagne, David Ames, Naoki Kaneko, Sam L. Gardener, Kevin Taddei, Binosha Fernando, Ian Martins, Prashant Bharadwaj, Hamid R. Sohrabi, Colin L. Masters, Belinda Brown, Ralph N. Martins

**Affiliations:** ^1^School of Medical Sciences, Sarich Neuroscience Research Institute, Edith Cowan University, Nedlands, WA, Australia; ^2^Department of Biomedical Sciences, Faculty of Medicine, Health and Human Sciences, Macquarie University, Sydney, NSW, Australia; ^3^Center for Development of Advanced Medicine for Dementia, National Center for Geriatrics and Gerontology, Obu, Japan; ^4^Centre for Healthy Ageing, Health Futures Institute, Murdoch University, Murdoch, WA, Australia; ^5^Department of Nuclear Medicine and Centre for PET, Austin Health, Heidelberg, VIC, Australia; ^6^Department of Psychiatry, University of Pittsburgh, Pittsburgh, PA, United States; ^7^National Ageing Research Institute, Parkville, VIC, Australia; ^8^Academic Unit for Psychiatry of Old Age, St George's Hospital, University of Melbourne, Kew, VIC, Australia; ^9^Koichi Tanaka Mass Spectrometry Research Laboratory, Shimadzu Corporation, Kyoto, Japan; ^10^The Florey Institute, The University of Melbourne, Parkville, VIC, Australia; ^11^School of Psychiatry and Clinical Neurosciences, University of Western Australia, Crawley, WA, Australia

**Keywords:** Alzheimer's disease, amyloid, physical activity, *APOE* genotype, biomarkers

## Abstract

Previous studies have indicated that physical activity may be beneficial in reducing the risk for Alzheimer's disease (AD), although the underlying mechanisms are not fully understood. The goal of this study was to evaluate the relationship between habitual physical activity levels and brain amyloid deposition and AD-related blood biomarkers (i.e., measured using a novel high-performance mass spectrometry-based assay), in apolipoprotein E *(APOE)* ε4 carriers and noncarriers. We evaluated 143 cognitively normal older adults, all of whom had brain amyloid deposition assessed using positron emission tomography and had their physical activity levels measured using the International Physical Activity Questionnaire (IPAQ). We observed an inverse correlation between brain amyloidosis and plasma beta-amyloid (Aβ)_1−42_ but found no association between brain amyloid and plasma Aβ_1−40_ and amyloid precursor protein (APP)_669−711_. Additionally, higher levels of physical activity were associated with lower plasma Aβ_1−40_, Aβ_1−42_, and APP_669−711_ levels in *APOE* ε4 noncarriers. The ratios of Aβ_1−40_/Aβ_1−42_ and APP_669−711_/Aβ_1−42_, which have been associated with higher brain amyloidosis in previous studies, differed between *APOE* ε4 carriers and non-carriers. Taken together, these data indicate a complex relationship between physical activity and brain amyloid deposition and potential blood-based AD biomarkers in cognitively normal older adults. In addition, the role of *APOE* ε4 is still unclear, and more studies are necessary to bring further clarification.

## Introduction

Alzheimer's disease (AD) is the most common form of dementia in older adults, and the number of affected individuals is set to escalate in the coming decades. Currently, there is no cure for AD, and available pharmaceutical therapies are focused on relieving the severity of associated symptoms. Extracellular plaques primarily comprising beta-amyloid (Aβ) deposits are one of the major hallmark pathologies of AD. The deposition of Aβ in the brain has been demonstrated to occur many years before the onset of clinical symptoms and contributes to neuronal death and loss of cognitive abilities (Villemagne et al., 2013). Thus, this research is focused on understanding and identifying interventions that can slow amyloid deposition and improve cognition in cognitively normal (CN) older adults who are currently at-risk for AD (based on high brain amyloid deposition) and in individuals with an AD diagnosis.

High levels of physical activity (PA) have been reported as one lifestyle factor that may protect against the development of AD pathology and, in addition, can slow brain atrophy and the associated progressive cognitive decline (Schultz et al., 2015; Delli Pizzi et al., 2020; Dougherty et al., 2021). Substantial evidence exists from animal studies to support the role of exercise in reducing brain Aβ levels, likely through multiple mechanisms. These mechanisms affect the Aβ production, by shifting the processing of amyloid precursor protein (APP) toward the non-amyloidogenic pathway *via* increases in a disintegrin and metalloproteinase (ADAM10) and reductions in beta-site APP cleaving enzyme (BACE). In addition, exercise in animal models has also demonstrated increased Aβ catabolism by upregulating Aβ-degrading enzymes such as insulin-degrading enzyme and neprilysin, among others (Moore et al., 2016; Koo et al., 2017; Khodadadi et al., 2018; Brown et al., 2019; Zhang X. et al., 2019; Zhang X. L. et al., 2019). Building on this animal work, several reports from human studies have linked higher levels of PA with lower brain amyloid deposition, measured under positron emission tomography (PET) using amyloid-binding ligands (Liang et al., 2010; Brown et al., 2012, 2013; Okonkwo et al., 2014; Rabin et al., 2019). As expected, CSF-related biomarkers were also affected by PA in cognitively healthy adults (Law et al., 2018).

Based on the current literature, there appears to be individual variability in the relationship between PA and AD-related pathologies, with carriage of the apolipoprotein E (*APOE*) ε4 alleles, the greatest known genetic risk factor for sporadic (i.e., non-familial) AD. PA has been associated with lower brain amyloid (Head et al., 2012; Brown et al., 2013) and Tau burdens (Brown et al., 2018) and preserved cognitive functions (Jensen et al., 2019) to a greater extent in *APOE* ε4 carriers, compared with non-carriers in both healthy controls and patients with AD. However, studies that have reported higher PA levels to be associated with reduced dementia risk years later and have provided inconsistent results regarding whether ε4 carriers or non-carriers receive the greatest benefit (Podewils et al., 2005; Rovio et al., 2005).

In addition to amyloid burden measured *via* neuroimaging, PA is associated with AD-related blood biomarkers such as plasma Aβ measured by enzyme-linked immunosorbent assays (ELISA) (Baker et al., 2010; Brown et al., 2013; Stillman et al., 2017). More recently, a high-performance immunoprecipitation-mass spectrometry (IP-MS) assay quantifying plasma Aβ peptides was validated in two independent cohorts (Nakamura et al., 2018). This assay was able to differentiate between individuals with high brain amyloid deposition from those with low brain amyloid deposition. The high specificity and sensitivity are key features of this assay and may highlight differences in Aβ levels that could go undetected when using less sensitive assays. As a consequence, this more sensitive Aβ assessment could be used to more specifically evaluate the efficacy of medical/physical therapies with regard to blood-based biomarkers. As different techniques of Aβ measurement may yield different observations, the goal of this study was to assess the relationship between brain Aβ deposition, AD-related blood biomarkers (assessed with a high-performance mass spectrometry assay), and habitual PA levels. We also wanted to evaluate whether the association between PA and AD-related biomarkers was more marked in *APOE* ε4 allele carriers.

## Materials and Methods

### The AIBL Cohort and Procedures

The Australian Imaging, Biomarkers and Lifestyle (AIBL) study of aging was approved by the Human Research Ethics Committees of St. Vincent's Health, Hollywood Private Hospital, and Austin Health and Edith Cowan University (Australia). All methods were performed in accordance with the relevant guidelines and regulations. AIBL is a longitudinal study comprising older adults (age range of 64–88 years) who are CN, have mild cognitive impairment (MCI) or AD, and are evaluated every 18 months. A more detailed description of the recruitment process has been previously described (Ellis et al., 2009). In the AIBL cohort, more than 2,350 individuals have been enrolled to date, and all participants gave written and informed consent before participation. Participants attended the study site in the morning, after an overnight fast. Several physical parameters, such as weight, blood pressure, and pulse rate, were recorded, after which a fasting blood sample was collected for subsequent processing and analysis (Ellis et al., 2009). Cognitive and lifestyle evaluations were then performed, and diagnostic classifications (i.e., CN, MCI, or AD) were performed in accordance with the National Institute of Neurological and Communicative Diseases and Stroke/Alzheimer's Disease and Related Disorders Association (NINCDS-ADRDA) criteria by an expert clinical panel (Folstein et al., 1975; Mckhann et al., 1984; Saxton et al., 2000; Winblad et al., 2004). For this study, a total of 143 CN participants (Mini-Mental State Examination [MMSE]≥ 25) (Pangman et al., 2000) who were previously assessed for brain amyloidosis, PA, and blood biomarkers, assessed with the novel high-performance IP-MS assay, employed by Nakamura and colleagues (Nakamura et al., 2018), were included. We acknowledged that, however, the absence of brain Tau levels (either by imaging or biofluid) is a limitation of this study.

### Blood Collection and APOE Genotype

Plasma was isolated from whole blood collected in ethylenediaminetetraacetic acid (EDTA) tubes by centrifugation, aliquoted, and stored at −80°C. The *APOE* ε4 status was determined by genotyping cells from whole blood as previously described (Gupta et al., 2011).

### Measurement of Aβ Species

Plasma Aβ levels were measured using IP-MS to quantify Aβ-related peptides of different mass using matrix-associated laser desorption/ionization time of flight (MALDI-TOF) mass spectrometry after isolation and enrichment by immunoprecipitation from plasma. Briefly, 250 μl of plasma was mixed with an equal volume of Tris buffer [10 pM stable-isotope-labeled (SIL) Aβ_1−38_ peptide, 0.2% w/v *n*-dodecyl-β-d-maltoside (DDM), and 0.2% w/v *n*-nonyl-β-d-thiomaltoside (NTM)]. Normalization of the signal for all Aβ-related peptides was performed using the SIL-Aβ_1−38_ peptide as internal standard, while DDM and NTM were used for reducing nonspecific binding. Antibody beads were prepared by coupling monoclonal antibody 6E10 (BioLegend) directly to Dynabeads M-270 Epoxy and then used to immunoprecipitate plasma Aβ-related peptides and the internal standard by incubating them with plasma samples for 1 h. Elution of the peptides was performed using glycine buffer (pH 2.8) containing 0.1% w/v DDM. Upon the adjustment of the pH to 7.4 with Tris buffer, the immunoprecipitation was repeated, and the peptides were eluted with 5 mM HCl in 70% acetonitrile and applied on four wells of a 900 μm μFocus MALDI plateTM (Hudson Surface Technology) prespotted with α-cyano-4-hydroxycinnamic acid (CHCA) and methanediphosphonic acid (MDPNA). MALDI-linear TOF mass spectrometer (AXIMA Performance, Shimadzu/KRATOS) equipped with a 337 nm nitrogen laser in the positive ion mode was used to acquire mass spectra. The levels of plasma Aβ-related peptides were normalized with SIL-Aβ_1−38_ and used as plasma Aβ-related peptide levels. The reproducibility of the assay was verified using human EDTA plasma. The intra- and interday assay coefficients of variance (CVs) obtained for Aβ_1−40_ were 4.2–4.7% (*n* = 5) and 3.2–6.8% (*n* = 3), respectively; for Aβ_1−42_, the CVs were 6.8–7.8% and 1.6–7.7%, respectively, and for APP_669−711_, the CVs were 2.9–8.2% and 4.7–10.7%, respectively, supporting the reliability of the measurements. A more detailed description of the mass spectrometry methods is reported elsewhere (Nakamura et al., 2018).

### Imaging Data

All participants within the current study underwent a PET scan with either Pittsburgh compound B (^11^C-PiB), flutemetamol (^18^F-FLUTE), or florbetapir (^18^F-FBP) to measure brain amyloid load (Pike et al., 2007). The PET methodology for each tracer has been described elsewhere (Rowe et al., 2010; Vandenberghe et al., 2010; Wong et al., 2010). For the semiquantitative analysis, a standardized uptake value (SUV) was obtained from cortical and subcortical brain regions and then related to the SUV of the recommended reference region of each tracer to generate a tissue ratio termed the SUV ratio (SUVR). For PiB, the SUVs were normalized to the cerebellar cortex; for flutemetamol, the SUVs were normalized to the whole cerebellum, and for florbetapir, the SUVs were normalized to the pons (Clark et al., 2011; Lundqvist et al., 2013). For the combination of data from different PET tracers, the Before the Centiloid Kernel Transformation (BeCKeT) values were used, which represent a linear transformed standardization of FLUTE and FBP SUVR onto “PiB-like” SUVR (Villemagne et al., 2014). The cutoff value used to define brain amyloidosis was 1.4, such that participants considered amyloid negative (Aβ-) had a BeCKeT SUVR score <1.4 and those considered amyloid positive (Aβ+) had a BeCKeT SUVR score ≥ 1.4.

### Measurement of Physical Activity

Levels of PA were measured using the International Physical Activity Questionnaire (IPAQ) (Craig et al., 2003). The IPAQ is a subjective questionnaire that relies on participants to recall their PA from the previous 7 days. It is composed of 4 sections, namely, work activity, transportation activity, housework, and leisure-time activity. A metabolic equivalent score (MET) was associated with each question, and the total of the MET score was then assessed by multiplying the MET scores by the number of minutes per week spent participating in that activity to produce a 7-day activity score (i.e., METs min/week). We excluded questionnaires in which reported PA levels were two SDs above or below the mean, as well as incomplete questionnaires. The IPAQ has been validated in several studies indicating that the questionnaire is suitable for the measurement of PA (Craig et al., 2003; Hagstromer et al., 2006). Based on the standard IPAQ scoring instructions, participants within this study were divided into individuals with low-to-moderate level physical activity (LMPA; combined due to small number in low group) or high physical activity (HPA), using the same parameters described elsewhere (Brown et al., 2018). Our analysis used self-reported levels of PA, and although we acknowledged self-report can be erroneous, the IPAQ is a validated tool, and within the AIBL study cohort, we have reported associations between self-reported IPAQ data and measures of objective PA using actigraphy (Brown et al., 2012).

### Statistical Analysis

Descriptive statistics including means and SDs or proportions were calculated for LMPA and HPA groups, with comparisons employing independent sample *t*-tests or χ^2^ tests as appropriate. Linear models were employed to compare continuous variables (i.e., Aβ_1−40_, Aβ_1−42_, APP_669−711_, Aβ_1−40_/Aβ_1−42_ ratio, and APP_669−711_/Aβ_1−42_ ratio) between categories, corrected for covariates age and sex. Response variables were log transformed as necessary to better approximate normality and variance homogeneity. The composite *z*-score was also calculated and used in linear model analyses (*z*-score: average of Aβ_1−40_/Aβ_1−42_ ratio and APP_669−711_/Aβ_1−42_ ratio individual *z*-scores [(*z*-score Aβ_1−40_/Aβ_1−42_ ratio + *z*-score APP_669−711_/Aβ_1−42_ ratio)/2]). Analyses were also run stratifying the cohort based on *APOE* ε4 carriage (ε4–/ε4+) and brain amyloid status (Aβ-/Aβ+). Associations between continuous variables were assessed using linear regression and partial correlation, with corrections for sex, age, and *APOE* ε*4* allele carriage status. A *p*-value < 0.05 was regarded as significant. Analyses were carried out using the SPSS version 25 software (Chicago, IL, USA).

## Results

Demographic details of the study participants are summarized in Table 1. No significant differences in age, sex, brain amyloid deposition, *APOE* ε4 carriage, and tracer used were found between participants with LMPA and HPA levels.

**Table 1:** Demographic information of study participants based on self-reported physical activity groups.

	**Total sample (*n =* 143)**	**LMPA (*n* = 65)**	**HPA (*n* = 78)**	***p***
Age (years, mean ± SD)	74.0 ± 5.5	73.9 ± 5.5	74.0 ± 5.5	0.912
Sex (*n* M/F)	77/66	32/33	45/33	0.312
Brain Aβ status (*n* Aβ –/+)	81/62	36/29	45/33	0.782
*APOE* ε4 allele carriers (*n* –/+)	91/52	41/24	50/28	0.899
PiB/FLUTE/FBP (*n*)	60/39/44	26/19/20	34/20/24	0.871

*Demographic data of study participants were stratified by physical activity levels. Low-to-moderate physical activity (LMPA) and high physical activity (HPA) levels were assessed using the International Physical Activity Questionnaire (IPAQ). Brain amyloid deposition was measured using positron emission tomography. Values are expressed as mean ± SD or as the number of cases overall. A univariate analysis or χ^2^ analysis was used to assess differences between the LMPA and HPA groups*.

We evaluated the differences in plasma AD-biomarker levels (i.e., Aβ_1−42_, Aβ_1−40_, and APP_669−711_) between the PA groups in all participants and after stratifying by *APOE* ε4 carriage status or brain amyloid status (Table 2). Lower plasma Aβ_1−42_ levels were observed in the HPA group compared with those in the LMPA group, in all participants (*p* = 0.017 and *p* = 0.024, unadjusted and adjusted, respectively). After stratifying by *APOE* ε4 status or brain amyloid status, this difference was evident only in non-ε4 carriers (*p* = 0.003 and *p* = 0.004, unadjusted and adjusted, respectively) and in the Aβ- group (*p* = 0.014 and *p* = 0.012, unadjusted and adjusted, respectively). A total of 60 individuals (out of 81) who are brain Aβ- were also *APOE* non-ε4 carriers (out of a total of 91). This may explain why brain Aβ- and *APOE* non-ε4 carrier groups appear to have similar results.

**Table 2:** Comparison of plasma biomarkers between low-to-moderate physical activity (LMPA) and high physical activity (HPA) groups.

	**LMPA**	**HPA**	**p(F)**	**p^**a**^(F^**a**^)**
**A** **β_1−42_**				
(a) All	0.344 ± 0.087	0.312 ± 0.060	*0.017 (5.844)*	*0.024 (5.189)*
(b) ε4–	0.364 ± 0.090	0.316 ± 0.058	*0.003 (9.002)*	*0.004 (8.602)*
ε4+	0.311 ± 0.073	0.305 ± 0.064	0.772 (0.085)	0.986 (0.000)
(c) Aβ-	0.367 ± 0.091	0.324 ± 0.056	*0.014 (6.283)*	*0.012 (6.576)*
Aβ+	0.315 ± 0.074	0.295 ± 0.061	0.267 (1.257)	0.422 (0.655)
**A** **β_1−40_**				
(a) All	8.731 ± 2.165	8.054 ± 1.686	*0.043 (4.184)*	0.057 (3.698)
(b) ε4–	8.948 ± 2.362	7.944 ± 1.537	*0.020 (5.655)*	*0.019 (5.745)*
ε4+	8.362 ± 1.766	8.251 ± 1.937	0.770 (0.086)	0.760 (0.094)
(c) Aβ-	8.849 ± 2.375	7.902 ± 1.631	*0.043 (4.220)*	*0.031 (4.817)*
Aβ+	8.586 ± 1.904	8.262 ± 1.761	0.483 (0.499)	0.867 (0.043)
**APP**_**669−711**_				
(a) All	0.296 ± 0.061	0.270 ± 0.054	*0.006 (7.669)*	*0.007 (7.520)*
(b) ε4–	0.305 ± 0.064	0.259 ± 0.049	*<0.001 (15.512)*	*<0.001 (14.810)*
ε4+	0.282 ± 0.054	0.289 ± 0.060	0.628 (0.237)	0.543 (0.375)
(c) Aβ-	0.294 ± 0.064	0.261 ± 0.050	*0.012 (6.612)*	*0.012 (6.580)*
Aβ+	0.299 ± 0.059	0.282 ± 0.059	0.227 (1.487)	0.260 (1.294)

*Plasma Aβ_1−42_, Aβ_1−40_, and APP_669−711_ levels were compared between low-to-moderate physical activity (LMPA) and high physical activity (HPA) groups in all (a) participants, (b) participants stratified by apolipoprotein E (APOE) ε4 genotype status (ε4–/ε4+), and (c) brain amyloid status (Aβ-/Aβ+), using general linear models. Physical activity was measured by the International Physical Activity Questionnaire (IPAQ), and brain amyloid deposition was measured using positron emission tomography. Plasma Aβ_1−42_, Aβ_1−40_, and APP_669−711_ data were natural log transformed to better approximate normality and variance homogeneity. p^a^(F^a^) represents p-values adjusted for age and sex. P < 0.05 (italic) was considered significant. Data are presented in mean ± SD*.

Similarly, lower plasma Aβ_1−40_ levels were observed in the HPA group compared with the LMPA group, in all participants (*p* = 0.043, unadjusted and a trend toward statistical significance upon adjustment, *p* = 0.057). Further, after stratifying by *APOE* ε4 status or brain amyloid status, this difference was evident only in the non-ε4 carriers (*p* = 0.020 and *p* = 0.019, unadjusted and adjusted, respectively) and in the Aβ- group (*p* = 0.043 and *p* = 0.031, unadjusted and adjusted, respectively) (Table 2). Plasma APP_669−711_ was observed to be lower in the HPA group compared with that in the LMPA group, in all participants (*p* = 0.006 and *p* = 0.007, unadjusted and adjusted, respectively). After stratifying by *APOE* ε4 status or brain amyloid status, this difference was significant only in the non-ε4 carriers (*p* < 0.001 for both unadjusted and adjusted) and in the Aβ- group (*p* = 0.012 for both unadjusted and adjusted) (Table 2).

We have also assessed the effect of PA with regard to brain amyloidosis, and we did not find any significant effect of PA before any stratification. The same results were observed after stratification for *APOE* ε4 status or brain Aβ status (Supplementary Table 1).

In Table 3, the ratios of plasma APP_669−711_/Aβ_1−42_ and Aβ_1−40_/Aβ_1−42_ were also assessed with regard to PA and *APOE* ε4 status or brain Aβ status, and no differences between the PA groups were observed in the whole cohort, nor after stratifying by *APOE* ε4 status or brain Aβ status. We also assessed the composite *z* score for plasma ratios of APP_669−711_/Aβ_1−42_ and Aβ_1−40_/Aβ_1−42_, and we did not observe any significant differences related to different intensities of PA. Stratifying by *APOE* ε4 status or brain amyloid status, like in our previous analyses, did not result in significant composite *z*-score differences in any of the subgroups (Table 3).

**Table 3:** Comparison of plasma biomarkers ratios between low-to-moderate physical activity (LMPA) and high physical activity (HPA) groups.

	**LMPA**	**HPA**	**p(F)**	**p^**a**^(F^**a**^)**
**APP**_**669−711**_ **/** **A** **β_1−42_**				
(a) All	0.880 ± 0.148	0.874 ± 0.140	0.801 (0.064)	0.653 (0.203)
(b) ε4–	0.855 ± 0.140	0.826 ± 0.124	0.303 (1.073)	0.311 (1.039)
ε4+	0.924 ± 0.154	0.960 ± 0.127	0.355 (0.870)	0.556 (0.351)
(c) Aβ-	0.814 ± 0.130	0.809 ± 0.108	0.848 (0.037)	0.840 (0.041)
Aβ+	0.963 ± 0.128	0.963 ± 0.130	0.984 (0.000)	0.694 (0.157)
**Aβ_1−40_/Aβ_1−42_**				
(a) All	25.76 ± 4.16	25.97 ± 3.47	0.736 (0.114)	0.709 (0.140)
(b) ε4–	24.89 ± 4.07	25.28 ± 3.25	0.611 (0.261)	0.645 (0.124)
ε4+	27.24 ± 3.97	27.21 ± 3.58	0.977 (0.001)	0.659 (0.197)
(c) Aβ-	24.31 ± 3.90	24.43 ± 3.12	0.881 (0.022)	0.963 (0.002)
Aβ+	27.55 ± 3.81	28.08 ± 2.77	0.533 (0.394)	0.303 (1.079)
**Composite z-score**				
(a) All	−0.004 ± 0.882	0.003 ± 0.816	0.960 (0.003)	0.966 (0.002)
(b) ε4–	−0.208 ± 0.844	−0.256 ± 0.741	0.771 (0.085)	0.751 (0.101)
ε4+	0.344 ± 0.852	0.466 ± 0.744	0.582 (0.307)	0.534 (0.392)
(c) Aβ-	−0.427 ± 0.695	−0.429 ± 0.625	0.988 (<0.001)	0.927 (0.009)
Aβ+	0.522 ± 0.810	0.593 ± 0.665	0.703 (0.147)	0.720 (0.130)

*APP_669−711_/Aβ_1−42_, and Aβ_1−40_/Aβ_1−42_ ratios and the composite z-score of the same ratios were compared between low-to-moderate physical activity (LMPA) and high physical activity (HPA) groups in all (a) participants, (b) participants stratified by apolipoprotein E (APOE) ε4 genotype status (ε4–/ε4+), and (c) brain amyloid status (Aβ-/Aβ+), using general linear models. Physical activity was measured by the International Physical Activity Questionnaire (IPAQ), and brain amyloid deposition was measured using positron emission tomography. p^a^(F^a^) represents p-values adjusted for age and sex. P < 0.05 (italic) was considered significant. Data are presented in mean ± SD*.

However, levels of plasma Aβ_1−42_ were significantly lower (*p* = 0.013 and *p* = 0.031, unadjusted and adjusted, respectively), and the ratios APP_669−711_/Aβ_1−42_ and Aβ_1−40_/Aβ_1−42_ were significantly higher (APP_669−711_/Aβ_1−42_: *p* < 0.001 for both unadjusted and adjusted; Aβ_1−40_/Aβ_1−42_: *p* = 0.001 and *p* < 0.001, unadjusted and adjusted, respectively) in *APOE* ε4 carriers compared with the ε4 non-carriers (Table 4). Similarly, the levels of Aβ_1−42_ were significantly lower (*p* = 0.001 for both unadjusted and adjusted), and the ratios APP_669−711_/Aβ_1−42_ and Aβ_1−40_/Aβ_1−42_ were significantly higher (APP_669−711_/Aβ_1−42_: *p* < 0.001 for both unadjusted and adjusted; Aβ_1−40_/Aβ_1−42_: *p* < 0.001 for both unadjusted and adjusted) in the Aβ+ group compared with the Aβ- group (Table 4). More detailed analysis indicated that in most cases, the differences in Aβ_1−42_ levels and APP_669−711_/Aβ_1−42_ and Aβ_1−40_/Aβ_1−42_ ratios are affected by *APOE* genotype and are irrespective of the intensity of the PA (Supplementary Table 2).

**Table 4:** Comparison of the effect of APOE ε4 status and brain Aβ status on plasma Aβ_1−42_ levels and blood biomarker ratios.

***APOE*** **ε4 status**				
**Aβ_1−42_**	ε4–	ε4+	p(F)^#^	p^a^(F^a^)^#^
All	0.337 ± 0.077	0.308 ± 0.068	*0.013 (6.297)*	*0.031 (4.746)*
**APP_669−711_/Aβ_1−42_**	ε4–	ε4+	p(F)	p^a^(F^a^)
All	0.839 ± 0.131	0.943 ± 0.139	* <0.001 (19.936)*	* <0.001 (15.129)*
**Aβ_1−40_/Aβ_1−42_**	ε4–	ε4+	p(F)	p^a^(F^a^)
All	25.10 ± 3.62	27.22 ± 3.72	*0.001 (11.077)*	* <0.001 (14.582)*
**Brain Aβ** **status**				
**Aβ_1−42_**	Aβ-	Aβ+	p(F)^#^	p^a^(F^a^)^#^
All	0.343 ± 0.077	0.305 ± 0.068	*0.001 (11.733)*	*0.001 (11.876)*
**APP_669−711_/Aβ_1−42_**	Aβ-	Aβ+	p(F)	p^a^(F^a^)
All	0.811 ± 0.117	0.963 ± 0.128	* <0.001 (54.898)*	* <0.001 (54.199)*
**Aβ_1−40_/Aβ_1−42_**	Aβ-	Aβ+	p(F)	p^a^(F^a^)
All	24.38 ± 3.47	27.83 ± 3.28	* <0.001 (36.509)*	* <0.001 (36.304)*

*Plasma Aβ_1−42_ and the ratios APP_669−711_/Aβ_1−42_ and Aβ_1−40_/Aβ_1−42_ were compared with regards to APOE ε4 genotype status (ε4–/ε4+), and brain amyloid status (Aβ-/+) using general linear models. Brain amyloid deposition was measured using positron emission tomography. Plasma Aβ_1−42_ data were natural log transformed to better approximate normality and variance homogeneity (#). p^a^(F^a^) represents p-values adjusted for age and sex. P < 0.05 (italic) was considered significant. Data are presented in mean ± SD*.

The linear regression analysis indicated that the levels of plasma Aβ_1−42_ were significantly and negatively associated with brain amyloid deposition in the whole cohort and both PA groups (*p* < 0.001, *p* = 0.010 and *p* < 0.001 for the whole cohort, LMPA and HPA, respectively) (Table 5). These results were confirmed in partial correlation analyses upon correction for age, sex, and *APOE* ε4 status, (*p* = 0.001, *p* = 0.027, and *p* = 0.001 for the whole cohort, LMPA, and HPA, respectively) (Table 5). Conversely, no statistically significant associations were observed between brain amyloid and Aβ_1−40_ or APP_669−711_, regardless of whether the analysis was performed with or without adjustment for age, sex, and *APOE* ε4 status (Table 5).

**Table 5:** Correlation of brain amyloid load with blood biomarkers levels in the all-study participants and after stratification by PA levels.

		**All participants**		**LMPA**		**HPA**	
**Brain Aβ** **deposition (Ln Aβ)**		**β**	***p***	**β**	***p***	**β**	***p***
Plasma Aβ_1−42_ (Ln Aβ_1−42_)	Unadjusted	*−0.335*	*<0.001*	*−0.318*	*0.010*	*−0.408*	*<0.001*
	Adjusted*	*−0.290*	*0.001*	*−0.280*	*0.027*	*−0.389*	*0.001*
Plasma Aβ_1−40_ (Ln Aβ_1−40_)	Unadjusted	−0.016	0.850	−0.069	0.584	0.020	0.864
	Adjusted*	−0.007	0.935	−0.092	0.478	0.028	0.812
Plasma APP_669−711_ (Ln APP_669−711_)	Unadjusted	0.071	0.402	0.035	0.783	0.081	0.480
	Adjusted*	0.055	0.519	0.095	0.464	0.000	0.999

Linear regression was performed between brain Aβ deposition and plasma Aβ_1−42_, Aβ_1−40_, and APP_669−711_ in all participants and after stratification based on physical activity levels. Data were natural log transformed to better approximate normality and variance homogeneity. Physical activity was measured by the International Physical Activity Questionnaire (IPAQ), and participants were stratified based on low-to-moderate-level physical activity (LMPA) and high physical activity (HPA). P < 0.05 (italic) was considered significant. Adjusted^*^ represents analyses adjusted for age, sex, and APOE ε4-carrier status.

## Discussion

In this report, we assessed the association between PA and blood biomarkers (plasma Aβ_1−40_, Aβ_1−42_, APP_669−711_ levels) in CN older adults. Additionally, we examined how the carriage of the *APOE* ε4 allele (i.e., an important risk factor for sporadic AD) (Corder et al., 1993) affects the relationship between PA and AD biomarkers. We observed that (a) individuals reporting higher levels of PA had lower plasma AD biomarkers in *APOE* ε*4* noncarriers and brain Aβ deposition groups and (b) plasma biomarker ratios are associated with *APOE* ε*4* carrier status and with brain Aβ deposition status.

To date, the linkage between PA, risk of dementia, and *APOE* ε4 status are not clear. PA has shown to be inversely associated with brain amyloid deposition and to a greater extent in *APOE* ε4 carriers (Head et al., 2012; Brown et al., 2013); however, contradictory results have been reported when examining dementia risk as an outcome measure (Podewils et al., 2005; Rovio et al., 2005). Our results indicate that high PA has a trend-level association with lower brain amyloid levels only in individuals with high brain amyloidosis. Additionally, high PA is associated, albeit nonsignificantly, with lower SUVR in noncarriers of the *APOE* ε4 allele, while it was not a factor in *APOE* ε4 carriers. These data are in partial contradiction with previously published findings, also using AIBL study data (Brown et al., 2013), in which the effect of PA on brain amyloid deposition was restricted to *APOE* ε4 carriers. Such discrepancies may come from the fact that these analyses were performed on different numbers of healthy controls, which may affect the final results. The main reason for using a different cohort was our interest in evaluating the effect of PA on AD biomarkers when these were assessed using a more specific assay. Such biomarkers were assessed using a high-performance mass spectrometry analysis (Nakamura et al., 2018), which has the advantage of having a higher specificity and sensitivity compared with commercial ELISAs, which have been widely used for the analysis of Aβ_1−40_ and Aβ_1−42_. Utilizing this more sensitive and more specific assay for the measurement of Aβ_1−42_, Aβ_1−40_, and APP_669−711_, Nakamura et al. were able to identify individuals (i.e., CN, MCI, and AD) with aberrant brain amyloidosis. However, this study could only be performed using healthy controls from the AIBL cohort who had AD biomarkers assessed by this new technique, and while our results may suggest a novel approach, one of the limitations of this study is the size of the cohort used.

The analysis of our biomarkers indicated that higher PA was significantly associated with lower levels of Aβ_1−40_, Aβ_1−42_, and APP_669−711_ in CN ε4 non-carriers, while no relationship was observed in those carrying at least one *APOE* ε4 allele. It must be noted, however, that while Aβ_1−42_ levels were significantly higher in ε4 carriers, *APOE* ε4 status had no effect on Aβ_1−40_ and APP_669−711_ levels. These data are in accordance with other reports that show associations of higher PA with lower levels of plasma Aβ in humans and mouse models of AD (Baker et al., 2010; Stillman et al., 2017; Khodadadi et al., 2018), although our original report did not observe a similar association between plasma Aβ and PA (Brown et al., 2013). Our original report, however, indicated an effect of HPA on the Aβ_1−42_/Aβ_1−40_ ratio in *APOE* ε*4* noncarriers (Brown et al., 2013). While the size of the cohort may still be a limiting factor, the more sensitive Aβ assays may also play a role in justifying these discrepancies. Increased specificity and sensitivity may detect forms of Aβ that could go undetected in regular ELISA kits, and this could lead to assessing Aβ levels more accurately. This, in turn, would allow for more reliable analyses involving Aβ levels that could lead to a more appropriate assessment of biomarkers, as seen with the findings of Nakamura et al., where plasma Aβ and a related fragment reflected brain amyloidosis with high accuracy.

Additionally, an inverse correlation between brain amyloid deposition and Aβ plasma levels observed in AD participants within the AIBL cohort suggested that patients with AD with higher brain amyloid deposition had lower plasma Aβ_1−42_ levels (Lui et al., 2010). These results indicate that in AD, Aβ_1−42_ is sequestered in the brain in the form of amyloid plaques resulting in lower plasma Aβ_1−42_ levels. As data from this study indicated that increased PA was associated with lower levels of brain amyloid and plasma levels of Aβ_1−40_, Aβ_1−42_, and APP_669−711_, we then evaluated if such inverse correlation was retained. As shown, we have reported that there is a significant inverse correlation between brain amyloid and plasma Aβ_1−42_, which was not affected by the level PA. One possible mechanism could be that the main effect of PA was associated with lower brain amyloid and plasma Aβ_1−40_, Aβ_1−42_, and APP_669−711_ levels (in non-ε4 carriers only) and is more likely a consequence of a shift toward a non-amyloidogenic pathway, while it has no effect on the transport of Aβ through the blood-brain barrier into the circulation.

**Figure 1:**
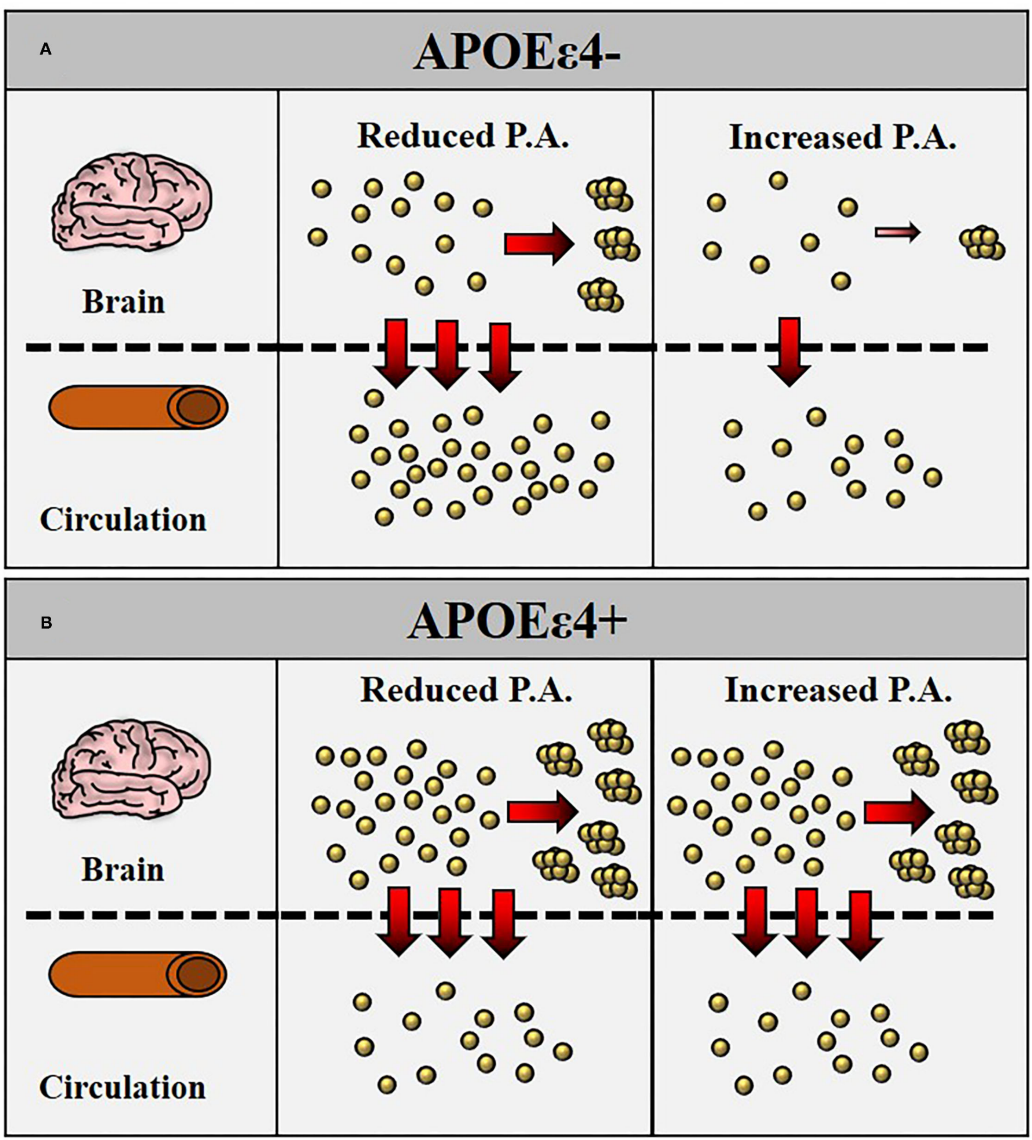
Schematic representation of how physical activity (PA) may reduce Aβ generation with consequently reduced transport of Aβ_1−42_ across the blood-brain barrier (BBB) into circulation. In apolipoprotein E *(APOE)* ε4– **(A)**, increased intensity of PA is followed by a reduced generation of Aβ_1−42_ with consequently reduced plaque formation in the brain and reduced transport of Aβ_1−42_ in the bloodstream. In *APOE* ε4+ **(B)**, the lower levels of Aβ_1−42_ in the bloodstream are a consequence of increased Aβ_1−42_ retention in the brain with greater plaque formation. The intensity of PA does not appear to affect Aβ_1−42_ plasma levels.

The ratio Aβ_1−40_/Aβ_1−42_ (or its inverse) and the ratio APP_669−711_/Aβ_1−42_ have also been indicated in previous reports as specific predictors of brain amyloidosis (Ovod et al., 2017; Nakamura et al., 2018; Chatterjee et al., 2019; Schindler et al., 2019) or AD (Lui et al., 2010). Our study, as all others, reported that these plasma biomarker ratios can significantly differentiate CN healthy controls with high brain amyloidosis vs. CN healthy controls with low brain amyloidosis; however, PA does not significantly alter these ratios. Similar results were obtained when stratified for *APOE* ε4 carrier status, where these ratios were significantly different in *APOE* ε4 carriers vs. noncarriers (due to the underlying *APOE* ε4 effect on plasma Aβ_1−42_), but these results were not affected by PA. A schematic representation of how PA can affect plaque formation and Aβ_1−42_ transport across blood-brain barrier (BBB) is illustrated in Figure 1.

To summarize, our data indicated that (a) PA was associated with lower levels of AD-related plasma biomarkers in healthy control *APOE* ε4 noncarriers and Aβ-individuals, (b) plasma levels of Aβ_1−42_, but not Aβ_1−40_ or APP_669−711_, inversely correlated with brain amyloidosis, and (c) PA was associated with lower brain amyloidosis in healthy controls at risk of AD, although the analysis approaches statistical significance. Although we have indicated that our study has some limitations, we have reported that PA influences AD biomarker levels, likely affecting the process underlying the amyloidogenic pathway. Further studies are, therefore, necessary to confirm the validity of our findings in a larger cohort and to determine the involvement of different levels of PA with regards to plasma AD biomarkers.

## Data Availability Statement

The original contributions presented in the study are included in the article/Supplementary Material, further inquiries can be directed to the corresponding author/s.

## Ethics Statement

The studies involving human participants were reviewed and approved by The Australian Imaging, Biomarkers and Lifestyle (AIBL) study of aging was approved by the Human Research Ethics Committees of St. Vincent's Health, Hollywood Private Hospital, Austin Health and Edith Cowan University (Australia). The patients/participants provided their written informed consent to participate in this study.

## Author Contributions

SR-S, VD, NK, and BB carried out experiments and collected data. SP, PC, and BB analyzed data and wrote the manuscript. MT, EH, SG, KT, PB, AN, SR-S, VV, CR, DA, BF, IM, HS, CM, and RM provided scientific input. RM supervised the project. All authors contributed to the article and approved the submitted version.

## Funding

Funding support from Australian Alzheimer's Research Foundation (AARF), Alzheimer's Australia (AA), the Science and Industry Endowment Fund, CSIRO, Brightfocus, USA and the WA Department of Health, as well as industry sources. We acknowledge the financial support of the CRC for Mental Health and the Cooperative Research Centre (CRC) program is an Australian Government Initiative.

## Conflict of Interest

NK was employed by Shimadzu Corporation. The remaining authors declare that the research was conducted in the absence of any commercial or financial relationships that could be construed as a potential conflict of interest.

## Publisher's Note

All claims expressed in this article are solely those of the authors and do not necessarily represent those of their affiliated organizations, or those of the publisher, the editors and the reviewers. Any product that may be evaluated in this article, or claim that may be made by its manufacturer, is not guaranteed or endorsed by the publisher.
