# Supplementary material for: The Association Between Alzheimer's Disease-Related Markers and Physical Activity in Cognitively Normal Older Adults
Source: Front Aging Neurosci. 2022 Mar 28;14:771214. doi: 10.3389/fnagi.2022.771214 (PMC8996810; doi:10.3389/fnagi.2022.771214)
Supplement: Supplementary file 1 [file Data_Sheet_1.docx]

**Supplementary Table 1. *Comparison of the effect of higher levels of PA on brain amyloid deposition, non-stratified and stratified for APOEε4 status and brain Aβ status***

| **SUVR** | LMPA | HPA | p(F) | p^a^(F^a^) |
| --- | --- | --- | --- | --- |
| (a) All | 1.54 ± 0.45 | 1.48 ± 0.35 | 0.420 (0.654) | 0.389 (0.747) |
| (b) ε4- | 1.46 ± 0.40 | 1.37 ± 0.26 | 0.289 (1.137) | 0.274 (1.215) |
| ε4+ | 1.69 ± 0.50 | 1.66 ± 0.41 | 0.949 (0.004) | 0.874 (0.026) |
| (c) Aβ- | 1.21 ± 0.10 | 1.23 ± 0.09 | 0.299 (1.093) | 0.210 (1.602) |
| Aβ+ | 1.96 ± 0.35 | 1.81 ± 0.29 | 0.081 (3.161) | 0.093 (2.924) |

Brain Aβ deposition levels were compared between low to moderate physical activity (LMPA) and high physical activity (HPA) groups in all (a) participants (b) participants stratified by Apolipoprotein E (APOE) ε4 genotype status (ε4-/ ε4+) and (c) brain amyloid status (Aβ-/ Aβ+) using general linear models. Physical activity was measured by the International Physical Activity Questionnaire (IPAQ) and brain amyloid deposition was measured using positron emission tomography. PiB data were natural log transformed to better approximate normality and variance homogeneity. p^a^(F^a^) represents p-values adjusted for age and sex. p<0.05 was considered significant. Data are presented in mean ± SD.

**Supplementary Table 2. *Comparison of the effect of APOE ε4 status and brain Aβ status on plasma Aβ_1-42_ levels and blood biomarker ratios between low to moderate physical activity (LMPA) and high physical activity (HPA) groups***

| ***APOE* ε4 status** | | | | |
| --- | --- | --- | --- | --- |
| **Aβ_1-42_** | ε4- | ε4+ | p(F) ^#^ | p^a^(F^a^) ^#^ |
| LMPA | 0.364 ± 0.090 | 0.311 ± 0.073 | *0.012 (6.721)* | *0.016 (6.116)* |
| HPA | 0.316 ± 0.058 | 0.305 ± 0.064 | 0.329 (0.966) | 0.743 (0.108) |
| **APP_669-711_/Aβ_1-42_** | ε4- | ε4+ | p(F) | p^a^(F^a^) |
| LMPA | 0.855 ± 0.140 | 0.924 ± 0.154 | 0.069 (3.421) | 0.101 (2.782) |
| HPA | 0.826 ± 0.124 | 0.960 ± 0.127 | *< 0.001 (20.711)* | *< 0.001 (14.518)* |
| **Aβ_1-40_/Aβ_1-42_** | ε4- | ε4+ | p(F) | p^a^(F^a^) |
| LMPA | 24.89 ± 4.07 | 27.24 ± 3.97 | *0.027 (5.144)* | *0.012 (6.651)* |
| HPA | 25.28 ± 3.25 | 27.21 ± 3.58 | *0.018 (5.876)* | *0.004 (8.881)* |
| **Brain Aβ status** | | | | |
| **Aβ_1-42_** | Aβ- | Aβ+ | p(F) ^#^ | p^a^(F^a^) ^#^ |
| LMPA | 0.367 ± 0.091 | 0.315 ± 0.074 | *0.011 (6.849)* | *0.008 (7.601)* |
| HPA | 0.324 ± 0.056 | 0.295 ± 0.061 | *0.019 (5.722)* | *0.029 (4.974)* |
| **APP_669-711_/Aβ_1-42_** | Aβ- | Aβ+ | p(F) | p^a^(F^a^) |
| LMPA | 0.814 ± 0.130 | 0.963 ± 0.128 | *< 0.001 (21.557)* | *< 0.001 (4.720)* |
| HPA | 0.809 ± 0.108 | 0.963 ± 0.130 | *< 0.001 (33.121)* | *< 0.001 (32.434)* |
| **Aβ_1-40_/Aβ_1-42_** | Aβ- | Aβ+ | p(F) | p^a^(F^a^) |
| LMPA | 24.31 ± 3.90 | 27.55 ± 3.81 | *0.001 (11.311)* | *0.004 (8.972)* |
| HPA | 24.43 ± 3.12 | 28.08 ± 2.77 | *< 0.001 (28.523)* | *< 0.001 (31.505)* |

Plasma Aβ_1-42_ and the ratios APP_669-711_/Aβ_1-42_ and Aβ_1-40_/Aβ_1-42_ were compared with regards to *APOE* ε4 genotype status (ε4-/ ε4+) and brain amyloid status (Aβ-/ Aβ+) in low to moderate physical activity (LMPA) or high physical activity (HPA) groups using general linear models. Brain amyloid deposition was measured using positron emission tomography. Plasma Aβ_1-42_ data were natural log transformed to better approximate normality and variance homogeneity (#). p^a^(F^a^) represents p-values adjusted for age and sex. p<0.05 was considered significant. Data are presented in mean ± SD.
